# Experience of Breast Augmentation in Pakistani Females

**Published:** 2017-09

**Authors:** Muhammad Ahmad

**Affiliations:** Department of Aesthetic Plastic Surgery, Islamabad Private Hospital, Islamabad, Pakistan

**Keywords:** Breast augmentation, Female, Pakistan

## Abstract

**BACKGROUND:**

Breast augmentation is an elective surgery used to increase the size of the breast in females. This study was undertaken to determine the breast augmentation in Pakistani females.

**METHODS:**

From 2006 to 2011, 43 female patients who underwent breast augmentation via infra-mammary, periareolar, trans-umbilical and fat transfer were enrolled. For augmentation, saline-filled or gel-filled implants were used in the supine position under general anesthesia and local infiltration of adrenaline (1:100,000 dilution) on each side. After the surgery, postoperative dressing was changed after 3-5 days and post-op bra was used for next 3-4 weeks. The patients were followed up for any complications too.

**RESULTS:**

The majority of patients (60.4%) were less than 30 years (mean age: 27.51 years). Most of patients (70%) had infra-mammary incision, 13.9% had periareolar, 9.3% had trans-umbilical and 4.7% had fat transfer. Saline-filled implants were used in 30.2%, whereas gel-filled implants in 69.8% of patients. Implant volume in infra-mammary, periareolar, trans-umbilical and fat transfer approaches was 278.9, 291.7, 277.5 and 325 mL, respectively. Only two cases of infection were recorded in early postop period. One patient responded to conservative treatment and in 2^nd^ patient, implants were removed. There was only one case of hypertrophic scar. No case of capsular contracture was seen. Quality of scar was satisfactory in infra-mammary and periareolar incisions. Changes in sensations were noted in 6 cases, 4 of them had periareolar incision.

**CONCLUSION:**

Properly performed breast augmentation results in restoration of physical and psychological well-being of the patient and less complication rates.

## INTRODUCTION

Breast augmentation is an elective surgery used to increase the size of the breast in females. It has gained worldwide acceptance in the last decades due to changing cultural trends, development of more modern implants, and refinements of the surgical techniques.^1^^,^^2^ In females, it is one of the most commonly performed procedure.^3^ The literature mentions various routes for implant placement, each having its merits and demerits.^4^^,^^5^ The infra-mammary approach allows the direct visualization of the dissected pocket. However, this technique leaves a visible scar within the inframammary fold.^1^^,^^5^

The periareolar approach is carried through the areolar-cutaneous junction and generally heals inconspicuously, if performed carefully. It gives limited exposure to the surgical filed, transection of the parenchymal ducts, increased risk of nipple-sensitivity changes and visible scar on the breast mound are the common drawbacks.^1^^,^^5^ The trans-axillary approach avoids any scar on the breast mound but requires more technical expertise and instrumentation.^1^^,^^4^ The trans-umbilical approach has a well-hidden incision scar, but it is the most difficult technique to learn and master.^1^^,^^4^^,^^5^ Fat transfer has also been attempted with high success rates.^6^ Various types of implants are available and can be placed in submuscular, subfascial, dual plane or subglandular.^7^^-^^9^ The current study was carried out to share the experience of breast augmentation in the private setup in Pakistani females.

## MATERIALS AND METHODS

The study was conducted in a private setup in female patients undergoing breast augmentation. The patients underwent breast augmentation via infra-mammary, periareolar, trans-umbilical and fat transfer. For augmentation, saline-filled or gel-filled implants were used. All the procedures were performed under general anesthesia. The careful history and examination was undertaken. The patients were photographed and decision about the implant size was planned in the pre-operative consultation. The procedures were performed with the patient in the supine position. The local infiltration of adrenaline (1:100,000 dilution) was carried out on each side.

**Fig. 1:**
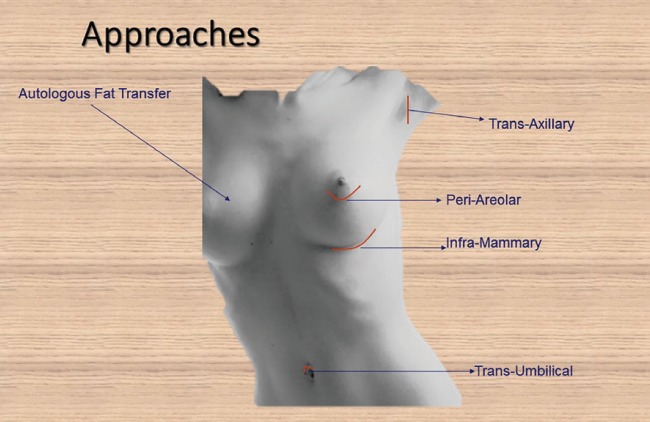
Available Routes for Breast Augmentation

For infra-mammary approach, the incision was located in the infra-mammary crease. Care was taken to position the resultant scar to be close to the crease and not allowed to ride on the breast mound. The subfascial plane was created and the haemostasis was secured. The size of the pocket was assessed and any necessary alteration was made (Figure 1). In periareolar approach, the incision was placed at the junction of skin and areola. Care was taken not to extend the incision beyond the 2/3^rd^ of the hemisphere of the areola and not to incise the skin adjacent to the areola. 

In trans-umbilical approach, special instruments were used to dissect the tunnel from the umbilicus towards the breast. Special dissectors were used to dissect the pocket. The saline-filled implants were used. For autologous fat transfer to the fat, the fat was harvested from the abdomen using fine cannulas. The fat was centrifuged and pure fat was re-injected into the breast in different planes using small (<2 mm) micro-cannulas. After the surgery, postoperative dressing was changed after 3-5 days and post-op bra was used for the next 3-4 weeks. The patients were followed up and any complication arising was noted and managed accordingly.

## RESULTS

The present study was conducted from 2006 to 2011. A total of 43 patients underwent the breast augmentation. The mean age of the patients was 27.51 years (range: 18–41 years). Majority of the patients (60.4%) were less than 30 years old including <20 years: 13.9%, 21–29 years: 46.5%, 30–39 years: 34.9%, and >40 years: 4.7%. Most the patients (70%) had infra-mammary incision, 13.9% had periareolar, 9.3% had trans-umbilical and in 4.7% of patients, fat transfer was performed. 

Saline-filled implants were used in 30.2%, whereas gel-filled implants were used in 69.8% of patients. The average implant volume of 278.9 mL (range: 145-425 mL) was used in the patients undergoing infra-mammary approach. The average implant volume of 291.7 mL (range: 175-345 mL) was used in periareolar approach, whereas the average volume was 277.5 mL (range: 255-305 mL) in trans-umbilical approach. The average volume of fat transfer was 325 mL (range: 300-350 mL). All the patients were followed up regularly. 

**Fig. 2:**
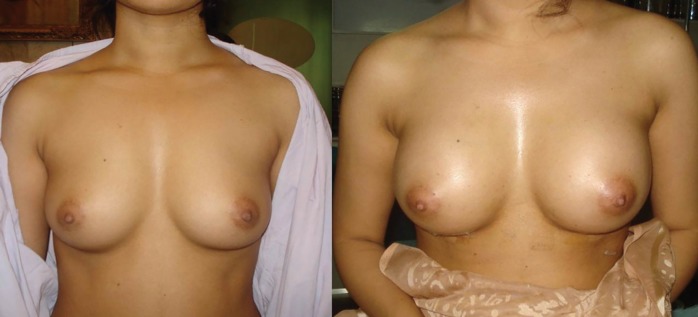
Trans-umbilical augmentation.

**Fig. 3:**
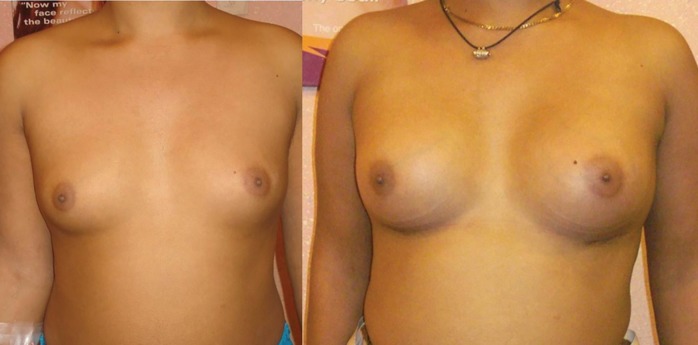
Infra-mammary augmentation

Altered sensations, infection, hypertrophic scar, capsular contracture, implant removal were noted in 13,8%, 6.4%, 2.3%, 2.3%, 2.3% of patients, respectively. Keloid, mismatch of size, lateral displacement were not seen in any of cases. The overall quality of scar was satisfactory in infra-mammary and periareolar incisions. Case 1 was a 25 years old female wanted an increase in the breast size. She underwent trans-umbilical approach and a saline-filled implant of 255 mL was used. The patient was satisfied with the outcome. (Figure 2). Case 2 was a 33 years old female who underwent gel-filled augmentation via infra-mammary approach. She underwent 345 mL implant and was satisfied with the result of surgery and the quality of the scar (Figure 3).

## DISCUSSION

**Fig. 4:**
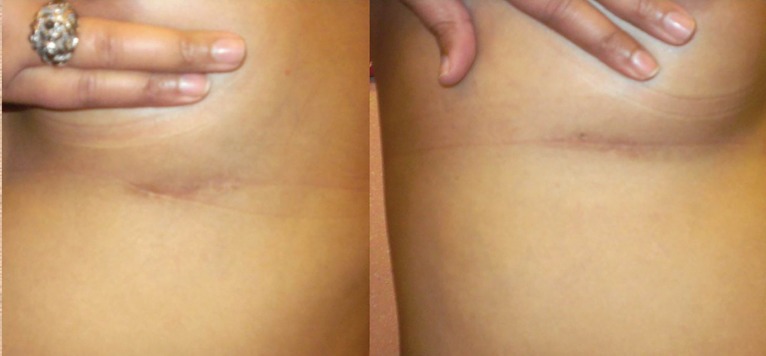
Scars quality of infra-mammary incisions.

Today breast augmentation is performed all over the world since its introduction in 1960’s by Cronin.^10^ The main approaches for breast augmentation remain the same with the introduction of fat transfer which has revolutionized the practice of breast augmentation. The infra-mammary approach is probably the most widely used. It gives a direct look in the dissection pocket and the scar can be minimized by using saline implants. Similarly, anatomical/tear drop implants can be used which have more patient satisfaction.^11^ The pocket of placement can also be changed. The main drawback of the techniques is a permanent scar (Figure 4). The periareolar technique is more difficult to perform. The approaches can be through the breast tissue or it can be extra glandular.^4^^,^^12^

The limitations are the permanent scar on the NAC-skin juncture which is the most prominent part of the breast. It may be unacceptable to many patients and secondly the limitation of insertion of larger implant. This problem can be handled by using saline-implants. The trans-axillary approach is more difficult to perform. Various instruments have been devised to aid the dissection of pocket and easy insertion of implant.^13^ The scar is very well-hidden but the learning curve is long. It can be performed by using endoscope as well.^14^ The trans-umbilical is the most difficult to perform and master. The technique can be performed with or without the use of endoscope.^15^^,^^16^ It gives a virtually scar-free augmentation with the scar well hidden in the umbilicus. However, revision surgery is not possible through this technique. The learning curve is the longest and only saline-implants can be used. 

Pakistan is a developing country. The norms are strict which restrict the females from getting personal aesthetic procedures. However, the modernization and education of the society has resulted in the change in behaviour towards the cosmetic surgery procedures. No published data on breast augmentation in Pakistani population is available to date. In the present study, 43 patients underwent cosmetic breast augmentation in a single centre which is very less as compared to the total number of surgeries performed by some others abroad.^17^^,^^18^ But the study is comparable to the similar studies carried out in the neighbor countries.^19^^,^^20^

The most important point in the present study was the lower complication rate. Hypertrophic scar was seen in only one patient. Capsular contracture was noted only in one case. Altered sensations were only felt by six patients. The overall satisfaction rate was over 80% (36 out of 43). The bathing of the pocket with povidine-iodine solution for 10 minutes was carried out in the patients in the current study, whereas the funnel use is reported to have lower capsular contracture rates.^21^

Fat transfer was used as a primary procedure and no case was done after implant removal because of the chances of more fibrosis and increased amount of fat required to fill up the cavity, which is contrary to a previous observation.^22^ As the implant actually exerts pressure on the breast parenchyma which decreases and ultimately requires more volume of fat to fill the cavity.^23^ We can conclude based on our findings that the breast augmentation resulted in restoration of physical and psychological well-being of the patients, even the techniques should be attempted to decrease the complication rates too. 

## CONFLICT OF INTEREST

The authors declare no conflict of interest.
